# Liver Fibrogenesis in Non-Alcoholic Steatohepatitis

**DOI:** 10.3389/fphys.2012.00248

**Published:** 2012-07-11

**Authors:** Zhaolian Bian, Xiong Ma

**Affiliations:** ^1^Division of Gastroenterology and Hepatology, Shanghai Institute of Digestive Disease, Renji Hospital, Shanghai Jiao-Tong University School of MedicineShanghai, China; ^2^Key Laboratory of Gastroenterology and Hepatology, Ministry of Health, Shanghai Jiao-Tong UniversityShanghai, China

**Keywords:** non-alcoholic fatty liver disease, hepatic stellate cell, hepatic fibrosis, cirrhosis

## Abstract

Non-alcoholic fatty liver disease (NAFLD) is emerging as one of the most common chronic liver diseases in developed western countries. Non-alcoholic steatohepatitis (NASH) is the most severe form of NAFLD, and can progress to more severe forms of liver disease, including fibrosis, cirrhosis, and even hepatocellular carcinoma. The activation of hepatic stellate cells plays a critical role in NASH-related fibrogenesis. Multiple factors, such as insulin resistance, oxidative stress, pro-inflammatory cytokines and adipokines, and innate immune responses, are known to contribute to the development of NASH-related fibrogenesis. Furthermore, these factors may share synergistic interactions, which could contribute to the process of liver fibrosis. Given the complex etiology of NASH, combined treatment regimes that target these different factors provide potential treatment strategies for NASH-related liver fibrosis.

## Introduction

Non-alcoholic fatty liver disease (NAFLD), affects one-third of adults and an increasing percentage of children in developed countries (Cohen et al., 2011). The disease spectrum of NAFLD includes simple steatosis, which is relatively benign, non-alcoholic steatohepatitis (NASH), NASH-related hepatic fibrosis, and cirrhosis (Jou et al., 2008). The disease begins with the aberrant accumulation of triglycerides in the liver, resulting in simple steatosis which is described as the benign form of NAFLD; most patients who develop steatosis are stable and further disease does not develop. However, some individuals progress to NASH, the severe form of NAFLD (Cohen et al., 2011). NASH is characterized by hepatocellular ballooning, lobular inflammation and hepatic fibrosis, besides steatosis (Brunt, 2004; Kleiner et al., 2005; Farrell and Larter, 2006). In NASH, 5–8% patients will develop cirrhosis within 5 years, and up to 20% of patients with NASH progress into cirrhosis in the end (Krawczyk et al., 2010). NASH is considered to be the most important subcategory of NAFLD, and has the largest influence on the prognosis of NAFLD. Characterizing the mechanisms of hepatic fibrogenesis in NASH is critical for preventing disease progression and improving the prognosis of patients with NAFLD. Similar to liver fibrosis caused by hepatitis B virus (HBV) or hepatitis C virus (HCV) infection, the activation of hepatic stellate cells (HSCs) is critical in hepatic fibrogenesis. The specific factors involved in the pathogenesis of NAFLD, such as insulin resistance, oxidative stress, pro-inflammatory cytokines, adipocytokines, and the innate immune response, may also contribute to disease progression and the development of NASH-related hepatic fibrogenesis. Understanding NASH-related hepatic fibrogenesis is an important research area and will be valuable for identifying potential therapeutic targets to prevent the progression of NAFLD to NASH and more severe disease.

## Activation of Hepatic Stellate Cells

Hepatic fibrosis, which is characterized by the excessive deposition of extracellular matrix (ECM) proteins, is considered to be a wound-healing process that results from a variety of chronic stimuli (Tsukada et al., 2006), such as viral hepatitis, NASH, or alcoholic liver disease. In adult NASH-related fibrosis, ECM is deposited primarily in the zone three perisinusoidal space of Disse, and then spreads to surround hepatocytes and thicken the space of Disse; forming characteristic “chicken-wire” fibrosis. Eventually, the pericentral fibrosis forms septa to isolate regenerating nodules (Law and Brunt, 2010; Pinzani, 2011).

The normal liver is composed of hepatocytes and non-parenchymal cells, which include kupffer cells, sinusoidal endothelial cells, and HSCs. HSCs are the major source of ECM in the fibrotic liver (Vera and Nieto, 2006). Normally, HSCs maintain a quiescent state and store a large amount of vitamin A. However, when the liver is injured HSCs undergo a phenotypic transition from a quiescent to activated phenotype. Accompanying this phenotypic transition, vitamin A is lost from the HSC, while the expression of smooth muscle α-actin (α-SMA) is increased. After activation, the proliferation of HSCs is increased, and their gene expression profile is altered, especially the expression of type I and III collagen (Tsukada et al., 2006). In addition to the proliferation and secretion of collagen, the contraction of activated HSCs is greatly strengthened, which could result in portal hypertension in patients with hepatic fibrosis (Tsukada et al., 2006). In addition to the transforming growth factor (TGF)-β signaling pathway, which is known to play major role in the activation of HSCs in liver fibrosis, many other signaling pathways are implicated in liver fibrosis in NAFLD, such as the hedgehog (Hh), PI3K/AKT, and JAK/STAT signaling pathways. Although the role of HSC activation in NAFLD has not been clarified completely, several studies have reported increased HSC activation in NASH (Kaji et al., 2011). The well-known role of HSCs in the pathogenesis of liver fibrosis suggests that they may play key role in NASH-related hepatic fibrosis, in which ECM deposition in the pericellular space forms a characteristic “chicken-wire” pattern (Marra et al., 2005).

## Insulin Resistance

Insulin resistance plays key role in pathogenesis of NAFLD, especially in hepatic steatosis (Krawczyk et al., 2010). Genetic polymorphisms and acquired factors contribute to insulin resistance (Williams et al., 2011). Several studies have demonstrated that insulin resistance is associated with NAFLD (Chitturi et al., 2002; Bloom et al., 2008; Fracanzani et al., 2008). Serum levels of insulin and glucose are increased in either genetic or acquired insulin resistance. Insulin resistance also has effects on HSCs, which play a key role in liver fibrosis (Rombouts and Marra, 2010). Insulin itself promotes mitogenesis of HSCs, mainly through binding to insulin receptors and the receptors for insulin-like growth factor-I. Glucose is also thought to significantly increase the expression of connective tissue growth factor, and slightly increase type I collagen expression in HSCs, both of which participate in NASH-related fibrogenesis (Paradis et al., 2001). In humans, insulin resistance is closely associated with advanced stages of fibrosis in patients with NAFLD (Bugianesi et al., 2004). In contrast, insulin sensitizers, such as pioglitazone (Promrat et al., 2004; Sanyal et al., 2004; Aithal et al., 2008), rosiglitazone (Ratziu et al., 2008), and metformin (de Oliveira et al., 2008), can attenuate NASH-related hepatic fibrosis. These data suggest that insulin resistance plays an important role in NASH-related fibrogenesis.

## Oxidative Stress

Oxidative stress reflects an imbalance between pro-oxidants and anti-oxidants with increased reactive oxygen species (ROS), (Chalasani et al., 2004) or decreased anti-oxidants (Koek et al., 2011). The generation of oxidative stress in NAFLD is associated with mitochondria, peroxisomes, and lipid peroxidation (Koek et al., 2011). In the context of NASH, it is known that oxidative stress induces the activation of HSCs (Guimaraes et al., 2006). For example, ROS can induce α-SMA, type I collagen and MMP-2 expression in HSCs via the p38/MAPK signaling pathway (Ikeda et al., 2011; Li et al., 2011). Furthermore, CYP2E1, which plays a key role in the generation of oxidative stress in NAFLD, activate HSCs, and increase the secretion of type I collagen; moreover, anti-oxidants and CYP2E1 inhibitors could block these effects (Urtasun et al., 2008). NADPH, which is present in many kinds of cells in the liver, such as kupffer cells, hepatocytes, and HSCs, participates in liver fibrosis (De Minicis et al., 2006, 2010). This is linked to the renin–angiotensin system, which also plays an important role in liver fibrogenesis through the activation of NADPH oxidase (Bataller et al., 2005). More importantly, anti-oxidants, such as vitamin E and astaxanthin, can alleviate NASH-related fibrogenesis, which suggests oxidative stress plays a role in NASH-related fibrogenesis (Sanyal et al., 2004; McCarty, 2011).

## Adipokines and Pro-Inflammatory Cytokines

Adipocytokines, which specifically refer to adipose tissue-derived cytokines, are composed of various factors secreted primarily by adipocytes, as well as inflammatory cells, including macrophages, and other infiltrating monocytes (Marra and Bertolani, 2009). Examples of adipokines include adiponectin, leptin, resistin, tumor necrosis factor-α (TNF-α), interleukin-6 (IL-6), and more recently discovered adipokines, such as visfatin, chemerin, vaspin (Kukla et al., 2011). It is thought that adipokines affect not only lipid metabolism, but also inflammatory and fibrotic processes in NAFLD (Marra and Bertolani, 2009).

Adiponectin is present in multimeric complexes in the plasma, and assembles into adiponectin trimers, hexamers, and 12- and 18-mers by means of its collagen domain (Rombouts and Marra, 2010). The effects of adiponectin are mediated by the two kinds of receptors, AdipoR2 and AdipoR1, which are primarily expressed in the liver and skeletal muscle, respectively (Marra et al., 2011). AdipoR2 expression was significantly decreased in rats fed a high-fat (HF) and cholesterol rich diet to induce inflammation and fibrosis in the liver, suggesting that AdipoR2 plays a major role in NAFLD (Matsunami et al., 2010). Several studies have also demonstrated that adiponectin has antifibrogenic effects in liver injury, and adiponectin deficiency exacerbates hepatic fibrosis induced by carbon tetrachloride (CCl_4_) in mice. *In vitro*, adiponectin suppresses HSC proliferation and migration, and attenuates the gene expression stimulated by TGF-β1 which is one of the most important pro-fibrogenic cytokines in liver injury induced by virus, NASH, and alcohol (Kamada et al., 2003). In adiponectin knockout mice fed a HF-diet, the pericellular fibrosis was more severe compared with WT mice (Asano et al., 2009). Similar results appeared in adiponectin knockout mice fed a choline-deficient l-amino acid-defined (CDAA) diet (Kamada et al., 2007). Furthermore, plasma adiponectin levels in patients with NASH are decreased, independent of the presence of obesity (Gastaldelli et al., 2010); however, another study found that adiponectin was elevated in patients with cirrhosis (Salman et al., 2010). Taken together, these data suggest that adiponectin is an important mediator of liver fibrosis.

Leptin is primarily secreted by adipocytes, but can also be produced by non-adipocyte cells, including HSCs (Zhang et al., 1994). The ob/ob mice, in which leptin is knocked out, developed less severe liver fibrosis induced by either CCl4 or thioacetamide (TAA), but when leptin levels were restored liver fibrosis was aggravated, suggesting that leptin is a potential pro-fibrogenic adipocytokine (Tsukada et al., 2006). Furthermore, it has been shown that leptin can promote the phenotypic transition of HSCs by activating the Hh pathway, altering gene expression programs that promote liver fibrosis. Meanwhile, the activation of the PI3K/AKT and JAK/STAT signaling pathways via binding to ObR (leptin receptor) contributes to the activation of the Hh pathway and mesenchymal gene expression, respectively (Choi et al., 2010). However, Cao et al. (2007) have reported that leptin could downregulate *MMP-1* gene expression in LX-2 cell line via the synergistic actions of the JAK/STAT pathway and the JAK-mediated ERK1/2 and p38 pathways. Recent studies found that the serum level of leptin was elevated in NASH patients (Uygun et al., 2000), and levels of soluble leptin receptor in serum were positively correlated with the stage of fibrosis in NAFLD patients (Medici et al., 2010).

Data related to visfatin, chemerin, and vaspin in NASH-related liver fibrosis are limited. The expression of visfatin in the liver was significantly higher in NAFLD patients with liver fibrosis and was positively correlated with the stage of fibrosis (Kukla et al., 2010a). It has also been separately shown that serum levels of chemerin and vaspin were both increased in patients with NAFLD (Kukla et al., 2010b; Yilmaz et al., 2011a), and the level of chemerin was modestly associated with liver fibrosis (Sell et al., 2010; Yilmaz et al., 2011b). The effects of chemerin and vaspin on liver fibrosis in NAFLD need to be studied in order to better understand their importance in the pathogenesis of NASH.

TNF-α is considered an important pro-inflammatory cytokine produced predominantly by the immune cells in the liver in NASH. IL-6, a multifunctional cytokine, promote insulin resistance (Kim et al., 2004), protect hepatocytes in steatotic liver by restraining oxidative stress and mitochondrial dysfunction (Cressman et al., 1996; El-Assal et al., 2004). Jin et al. (2006) reported that short-term IL-6 treatment protects mice from Fas-mediated liver injury and apoptosis, while result of long-term IL-6 treatment is paradoxical. These cytokines are involved in the transformation of HSCs into myofibroblasts, which contribute to the progression of liver fibrosis. TNF-α affects HSCs via binding to the TNF receptor-1, which is required for HSC proliferation and increasing MMP-9 expression (Tarrats et al., 2011). Serum levels of IL-6 in patients with NASH is associated with liver fibrosis (Lemoine et al., 2009). Taken together, these data suggest cytokines may play roles in liver fibrosis in NAFLD, and may present as targets for the treatment of liver fibrosis.

## Toll-Like Receptors

The multiple parallel hits hypothesis was proposed recently by Tilg and Moschen (2010) to explain the pathogenesis of NASH. This hypothesis states that various parallel factors, including gut-derived and adipose tissue-derived factors contribute to the development of liver fibrosis in NAFLD. The endotoxin lipopolysaccharide (LPS), derived from bacteria cell walls in the gut is known to play a role in the development of liver inflammation and fibrosis (Day and James, 1998; Jou et al., 2008). LPS has its effect by binding to the pattern-recognition receptors, especially Toll-like receptor (TLR)-4, where it triggers multiple intracellular signaling pathways, and then amplifies and maintains the inflammatory and fibrogenic signals in the liver (Brun et al., 2005; Seki et al., 2007). In brief, LPS activates HSCs through binding to TLR4 on the cellular surface, this promotes HSC proliferation and collagen production. TLR9, another TLR, was reported to promote HSC activation and to upregulate collagen production *in vitro* (Watanabe et al., 2007). Recently, Miura et al. (2010) also showed that TLR9 knockout mice developed less steatohepatitis and liver fibrosis in a murine NAFLD model, through suppressing the IL-1β produced by kupffer cells.

## Natural Killer T Cells

Natural killer T (NKT) cells, a subset of lymphocytes that secretes not only Th1-type cytokines such as interferon-γ, but also Th2-type cytokines such as IL-4 (Hegde et al., 2010). Studies reported that the HF-diet mice induced NKT cell apoptosis in the liver, which resulted in the decrease of hepatic NKT cells (Li et al., 2005; Deng et al., 2009). Oral immune regulation may alleviate steatosis in ob/ob mice through increasing hepatic NKT cells (Elinav et al., 2006). However, the population of hepatic NKT cells in NAFLD patients is controversial. Kremer et al. (2010) reported that hepatic NKT cells were decreased in NASH patients, and was associated with worse degrees of steatosis grade. In contrast, Tajiri et al. (2009) found that NKT cells in the liver and peripheral blood was increased with increasing NAFLD activity score. Adler et al. (2011) reported that NKT cells in the liver and blood significantly increased in patients with moderate to severe steatosis. CD1d-knockout mice, lacking NK1.1+ T cells, developed minimal hepatic fibrosis following chronic TAA treatment, compared to wild type mice (Ishikawa et al., 2011). Recently, it was shown that activation of the Hh pathway lead to hepatic accumulation of NKT cells that may activate HSC cells, resulting in progression of liver fibrosis in NASH (Syn et al., 2010). These data suggest that NKT cells may play pivotal roles in pathogenesis of NAFLD, not only in inflammation and steatosis, but also in fibrosis.

## Nuclear Receptors

Nuclear receptors regulate the expression of genes via binding directly to DNA. Several nuclear receptors, such as retinoid acid receptors (RAR), retinoid X receptor (RXR), and peroxisome proliferator-activated receptors (PPARs), participate in the process of phenotypic transition from quiescent HSCs to activated myofibroblastic-like cells (Wagner et al., 2011).

PPARs also play a key role in HSC biology and fibrosis in NAFLD, especially PPARγ (De Minicis and Svegliati-Baroni, 2011). It is known than PPARγ plays a role in the maintenance of a quiescent HSC phenotype, and that PPARγ agonists suppress the fibrogenic potential of HSCs *in vitro* and *in vivo*; specifically, pioglitazone and rosiglitazone, two kinds of PPARγ agonists, have been shown to alleviate liver inflammation and fibrosis in murine NASH models (Polyzos et al., 2010; Nakagami et al., 2012). Furthermore, pioglitazone also decreased liver fibrosis in patients with NASH (Gastaldelli et al., 2010; Ratziu et al., 2010); though another study reported pioglitazone could decrease inflammation in liver, but did not affect liver fibrosis (Belfort et al., 2006).

The farnesoid X receptor (FXR), also known as the bile acid receptor, induces expression of the small heterodimer partner, which may induce *PPAR*γ gene expression and then inhibit the activation of HSCs (Fiorucci et al., 2004; Renga et al., 2011). GW4064, an agonist of FXR, has been shown to inhibit the transdifferentiation of HSCs, and reduce their contractile response to endothelin-1. (Li et al., 2010) The liver X receptors (LXRs) are members of the metabolic nuclear receptor family that plays roles in the regulation of cholesterol absorption, efflux, transport, and excretion, amongst others. Beaven et al. (2011) found that LXR ligands suppressed the activation of primary mouse stellate cells and expression of fibrosis-related genes, leading such ligands to be considered new antifibrogenic factors (Mallat and Lotersztajn, 2011).

## Animal Models of NASH-Related Fibrogenesis

Dietary models of NASH include methionine-and choline-deficient (MCD) diet, the F diet and atherogenic diets (Schattenberg and Galle, 2010). Mice or rats fed with a MCD diet develop hepatic steatosis, which then progresses into steatohepatitis, and eventually leads to pericellular fibrosis (George et al., 2003; Sahai et al., 2004). The MCD diet is the most commonly used animal model to study pathogenesis of NASH-related fibrogenesis. However, the pathogenesis of fibrosis in mice fed with the MCD diet, including weight loss, increased peripheral insulin sensitivity, and loss of white adipose tissue are not characteristic of NASH-related fibrogenesis in human beings (Rinella and Green, 2004; Leclercq et al., 2007). Mice fed with a CDAA diet, display a similar liver phenotype to mice fed a MCD diet, but without weight loss, which suggests that this may be a better model (Kodama et al., 2009). Although a HF-diet increases metabolic risk factors in mice or rats, such as obesity, glucose intolerance, and increased lipogenic transcription factors, it rarely progress into liver fibrosis; however, 46% of mice overfed with a HF-diet (using an intragastric feeding protocol), developed steatohepatitis, and sinusoidal and pericellular fibrosis (Deng et al., 2005), although the high mortality rate and requirement for technical expertise means that the application of this model is limited.

Genetic models of NAFLD, including ob/ob mice, db/db mice, fa/fa mice, *KK-Ay/a mice*, do not develop liver fibrosis, except for mice that overexpress of SREBP-1c (Halaas et al., 1995; Nakayama et al., 2007; Schattenberg and Galle, 2010). Recently, Ota et al. reported a combination of a genetic and feeding model, called the Otsuka Long-Evans Tokishima Fatty (OLETF) rat. When such rats is fed with a MCD diet or a fat-enriched MCD diet, they progress to severe liver fibrosis (Ota et al., 2007).

## Conclusion

**Figure 1:**
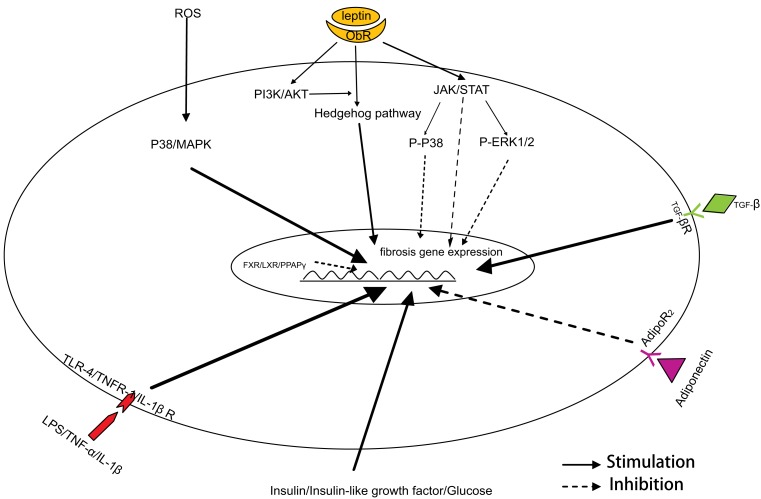
**Mechanism of HSC activation in NASH-related fibrogenesis**.

NASH currently represents one of the most prevalent liver diseases in humans, which is secondary to the increasing prevalence of obesity and the metabolic syndrome. It is well-known that the activation of HSCs is one of the critical events in NASH-related fibrogenesis. Insulin resistance, oxidative stress, pro-inflammatory cytokines, adipokines, and the innate immune response are involved in the phenotypic transition of HSCs (Figure 1), which then results in the development of NASH-related hepatic fibrogenesis. Of course, there are other factors we not mentioned here, such as endocannabinoid system and renin-angiotensin-aldosterone system. Because they are not as characteristic as insulin resistance and oxidative stress in NASH, although they play roles in NASH- related fibrogenesis. All these factors may interact with each other, and form a unique network that leads to the pathogenesis of liver fibrosis. Combined treatments targeted to these different factors are a feasible strategy in NASH-related liver fibrosis. In addition, an ideal animal model of NASH will help us to characterize the mechanisms of liver fibrosis in metabolic syndrome and to identify novel therapeutic approaches in the treatment of liver fibrosis.

## Conflict of Interest Statement

The authors declare that the research was conducted in the absence of any commercial or financial relationships that could be construed as a potential conflict of interest.
